# EGCG Maintains Th1/Th2 Balance and Mitigates Ulcerative Colitis Induced by Dextran Sulfate Sodium through TLR4/MyD88/NF-*κ*B Signaling Pathway in Rats

**DOI:** 10.1155/2017/3057268

**Published:** 2017-12-18

**Authors:** Xue Bing, Liu Xuelei, Dong Wanwei, Liang Linlang, Chen Keyan

**Affiliations:** ^1^Department of Endocrinology, General Hospital of Shenyang Military Area Command, No. 83 Wenhua Road, Shenyang, Liaoning 110016, China; ^2^Department of Laboratory, General Hospital of Shenyang Military Area Command, No. 83 Wenhua Road, Shenyang, Liaoning 110016, China; ^3^Department of Laboratory Animal Science, China Medical University, No. 77 Puhe Road, Shenyang North New Area, Shenyang, Liaoning 110122, China

## Abstract

**Objective:**

To observe the protective effect of epigallocatechin gallate (EGCG) on dextran sulfate sodium- (DSS-) induced ulcerative colitis in rats and to explore the roles of TLR4/MyD88/NF-*κ*B signaling pathway.

**Methods:**

Rat models of ulcerative colitis were established by giving DSS. EGCG (50 mg/kg/d) was given to assess disease activity index. HE staining was applied to observe histological changes. ELISA and qPCR detected the expression of inflammatory factors. Flow cytometry was used to measure the percentage of CD4^+^IFN-*γ*^+^ and CD4^+^IL-4^+^ in the spleen and colon. TLR4 antagonist E5564 was given in each group. Flow cytometry was utilized to detect CD4^+^IFN-*γ*^+^ and CD4^+^IL-4^+^ cells. Immunohistochemistry, qPCR, and western blot assay were applied to measure the expression of TLR4, MyD88, and NF-*κ*B.

**Results:**

EGCG improved the intestinal mucosal injury in rats, inhibited production of inflammatory factors, maintained the balance of Th1/Th2, and reduced the expression of TLR4, MyD88, and NF-*κ*B. After TLR4 antagonism, the protective effect of EGCG on intestinal mucosal injury was weakened in rats with ulcerative colitis, and the expressions of inflammatory factors were upregulated.

**Conclusion:**

EGCG can inhibit the intestinal inflammatory response by reducing the severity of ulcerative colitis and maintaining the Th1/Th2 balance through the TLR4/MyD88/NF-*κ*B signaling pathway.

## 1. Introduction

Ulcerative colitis (UC) is a refractory inflammatory disease of the large intestine [1], but the pathogenesis of UC is not yet clear. Currently, the treatment of UC includes surgical treatment and drug therapy. In addition to the routinely used corticosteroids and aminosalicylic acid [2], many new drugs including anti-tumor necrosis factor antibody are also widely used in clinical practice [3, 4]. Nevertheless, UC often relapses in a certain period of time after treatment, which brings serious economic and mental distress to patients and their families [5]. Therefore, understanding the pathogenesis of UC and identifying medications to effectively treat UC are in urgent need.

T cells occupy an important place in the immune system [6, 7]. Experiments have shown that local CD4^+^ T cells infiltrate and exhibit abnormal functional status in inflammatory bowel disease [8]. CD4^+^ T cells are divided into Th1 and Th2 cells according to their secretion of cytokines [9]. Under normal conditions, Th1 and Th2 in the body are in dynamic balance and regulate each other, so as to maintain the stability of the environment in the body. If Th1 and Th2 are out of balance, inflammation occurs in the tissues and organs and causes disease. It was found that the CD4^+^ T cells in UC mice produced large amounts of IL-4 and IL-5, and the single IFN-*γ* was normal or decreased [10], suggesting that the occurrence and development of UC were strongly associated with the imbalance of Th1 and Th2 cells.

Toll-like receptors (TLRs) are transmembrane protein family receptors that play a key role in nonspecific or innate immune defense [11]. TLR4 is a key element in the TLRs family. A previous study confirmed that, after external stimulation, the organism initiates the innate immune response, upregulates TLR4 expression, and activates NF-*κ*B via MyD88 dependent signaling pathway, thereby resulting in severe abnormality in intestinal mucosal epithelium [12]. Abnormal activation of TLR4/MyD88/NF-*κ*B signaling pathway in colonic mucosa of UC patients causes persistent aggravation of intestinal inflammation and becomes targets for drug therapy in UC patients [13].

Epigallocatechin gallate (EGCG) is the most abundant catechin in tea and is the main component of the biological activities of tea polyphenols [14]. Numerous studies have verified that EGCG has antioxidant and anti-inflammatory effects in cardiovascular diseases and lung, liver, and kidney diseases and has a protective effect in a variety of animal models of acute and chronic kidney disease [15, 16]. Therefore, this study sought to investigate the protective effect of EGCG on UC and to explore the possible molecular mechanism.

## 2. Materials and Methods

### 2.1. Experimental Animals and Group Assignment

Forty specific-pathogen-free male Sprague-Dawley rats weighing 260–289 g were purchased from Department of Laboratory Animals, China Medical University, China. This experiment was approved by the Institutional Animal Care and Use Committee of China Medical University (IACUC number 2015048). All rats were randomly assigned to sham surgery group (sham group; *n* = 10), DSS-induced UC group (UC group; *n* = 10), UC + EGCG group (EGCG group; *n* = 10), and UC + EGCG + TLR4 inhibitor E5564 group (TLR4 group; *n* = 10).

### 2.2. UC Models

Rat models of DSS-induced UC were established in accordance with a previous study [17]. The rats were allowed free access to solution containing 5% DSS (molecular weight 5000) for 7 days and then given distilled water for 14 days. After model establishment, the rats were intraperitoneally injected with EGCG 50 mg/kg/d, for 10 consecutive days.

### 2.3. Disease Activity Index (DAI)

According to body weight, stool, and blood in the stool, the rats were scored. Score 0 indicates no weight loss, normal stool, and no blood in the stool; score 1 indicates 1%–5% weight loss, loose stools, and fecal occult blood; score 2 indicates 5%–10% weight loss, loose stools, and fecal occult blood; score 3 indicates 10%–15% weight loss, watery stool, and gross blood stool; score 4 indicates more than 15% weight loss, watery stool, and gross blood stool.

### 2.4. Severity of Colonic Mucosal Injury

In accordance with Luketal's standards, score 0 indicates normal and no injury; score 1 indicates hyperemia and no ulcer; score 2 indicates hyperemia, bowel wall thickening, and no ulcer; score 3 indicates one small ulcer focus in 0-1 cm diameter; score 4 indicates big ulcer focus in 1-2 cm diameter and no bowel canal adhesion to the surrounding organs; score 5 indicates ulcer focus in 1-2 cm diameter, bowel thickening, and severe adhesion to adjacent organs.

### 2.5. Hematoxylin-Eosin Staining

All rats were sacrificed after EGCG withdrawal; rat colonic mucosa was fixed in 10% formaldehyde, decalcified, dehydrated, permeabilized, embedded in wax, and sliced into 5 *μ*m thick sections with a microtome. Sections were dewaxed with xylene, hydrated with absolute ethanol, and mounted with hematoxylin and eosin. Histological changes were observed under a microscope.

### 2.6. Enzyme Linked Immunosorbent Assay (ELISA)

ELISA kit (Cloud-Clone Corp., USA) was used to measure the changes in IL-2, IFN-*γ*, IL-4, and IL-10 in rat serum and tissue in each group in strict accordance with the instruction. Standard preparation (50 *μ*l) was added in the first and second wells, followed by multiple proportion dilution. Forty *μ*l sample diluent and 10 *μ*l sample (diluent : sample = 4 : 1) were added in each detected well and incubated at 37°C for 30 minutes. After washes with washing liquid for five times, secondary antibody (50 *μ*l) was added in each well and incubated at 37°C for 30 minutes. Chromogenic agents A and B (50 *μ*l for each) were added in each well and incubated at 37°C in the dark for 15 minutes. Termination solution (50 *μ*l) was added in each well. Microplate reader (Bio-Rad, USA) was used for detection. Standard curve was drawn and sample concentration was calculated.

### 2.7. Flow Cytometry for Determining CD4^+^IFN-*γ*^+^ and CD4^+^IL-4^+^ Lymphocytes in the Spleen

Mononuclear cells were isolated from the colon of rats. After counting, cells were incubated in a 5% CO_2_ incubator at 37°C for 24 hours. Nineteen hours later, cells in each well were treated with PMA (Sigma, P1585), ionomycin (Sigma, I-0634), and Brefeldin A (BD, 555029) to reach a final concentration of 10 ng/ml, 0.5 *μ*g/ml, and 1 *μ*l/ml. After stimulation, cells were placed in EP tube, incubated with FITC-anti-CD4 at room temperature in the dark for 30 minutes, washed with cell staining buffer, mixed with 500 *μ*l fixation/permeabilization solution (BD, 555028), and incubated at room temperature in the dark for 45 minutes. After being washed with 1x BD perm/wash buffer, cells were incubated with PE-anti-IL-4 and APC-anti-IFN-*γ* at room temperature in the dark for 45 minutes, washed with 1x BD perm/wash buffer, and resuspended with 300 *μ*l flow washing liquid. Samples were measured with flow cytometry and analyzed using FlowJo software.

### 2.8. Immunohistochemical Staining

Approximately 1 cm^3^ block of rat colon was fixed in 4% paraformaldehyde for 30–60 minutes and washed twice with PBS, each for 2 minutes. Tissue was dehydrated at 50°C, embedded in paraffin, and sliced into 4 um thick sections. The sections were placed on a slide and received treatment for adhesions. All samples were dewaxed, hydrated, treated with 3% H_2_O_2_ to deactivate endogenous enzyme, and immersed in 0.01 mol/L citrate for antigen retrieval. These sections were blocked with 5% bovine serum albumin for 20 minutes, incubated with primary antibody TLR4 (1 : 500), MyD88 (1 : 1000), and NF-*κ*B (1 : 1000) at 37°C for 1 hour, and washed two or three times with PBS. Subsequently, the sections were incubated with biotinylated goat anti-mouse IgG at 20–37°C for 20 minutes, washed four times with PBS, each for 5 minutes, visualized with 3,3′-diaminobenzidine, washed with distilled water, dehydrated, permeabilized, mounted with resin, and observed with a microscope.

### 2.9. Real-Time Polymerase Chain Reaction (PCR)

The colon was triturated and treated with Trizol reagent. RNA was precipitated, dried, and dissolved with 50 *μ*l DEPC-treated water. According to the manufacturer's instruction of RevertAid^TM^ First Strand cDNA Synthesis Kit (K1621, Thermo), the first strand of complementary DNA (cDNA) was produced by reverse transcription. SYBR Green (204054, Qiagen) was used for real-time fluorescence quantitative PCR. The relative gene expression data were analyzed with the 2^∧^(−Delta Delta CT) method. The primers were designed and synthesized with Sangon Biotech (Shanghai) Co., Ltd., China. The primers used for real-time PCR were listed in Table 1.

### 2.10. Western Blot Assay

The colonic tissue was treated with precooled lysate and centrifuged at 12000 rpm for 30 minutes. Total protein (the supernatant) was extracted and subjected to sodium dodecyl sulfate-polyacrylamide gel electrophoresis. The proteins were transferred onto the membrane by the semidry method. The membrane was blocked with confining liquid for 2 hours, incubated with primary antibodies TLR4 (1 : 500), MyD88 (1 : 1000), NF-*κ*B (1 : 1000), and GAPDH (1 : 2000) (Abcam, USA) at 4°C overnight, washed three times with TBST, and then incubated with secondary antibody for 1 hour. After four washes with TBST, the membrane was visualized with enhanced chemiluminescence reagent. Images were obtained using a gel imaging system. Gray value was read with Quantity One software.

### 2.11. Statistical Analysis

Data were analyzed using SPSS 19.0 software and expressed as mean ± SD. The difference between samples was compared using group *t*-test. The difference among groups was compared using one-way analysis of variance. For heterogeneity of variance, Tamhane-*t* or Dunnett-*T*3 test was used. A value of *p* < 0.05 was considered statistically significant.

## 3. Results

### 3.1. EGCG Lessens Colonic Mucosal Injury in UC Rats

**Figure 1:**
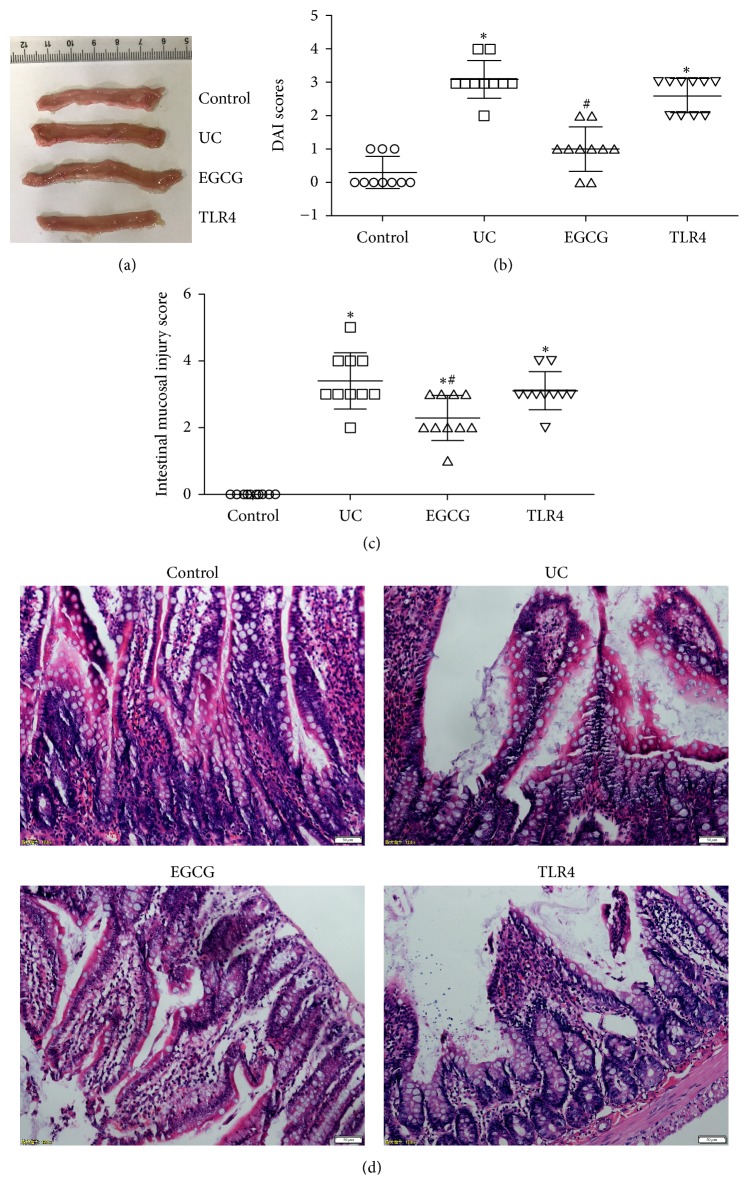
The UC model was established; the rats were intraperitoneally injected with EGCG 50 mg/kg/d for 10 consecutive days. The rat colonic tissue was collected, and colonic mucosal injury was observed (a). The rats were scored based on body weight, stool, and blood in the stool. (c) In accordance with Luketal's standards, colonic mucosal injury was scored. (d) The rat colonic tissue was collected, and the colonic pathological changes were observed after the HE staining (b). Data were collected from the control group, UC group, EGCG group, and TLR4 group (*n* = 10). Compared with control group, ^*∗*^*p* < 0.05. Compared with UC group, ^#^*p* < 0.05.

Compared with the sham group, DAI score was significantly higher in the UC group (*p* < 0.05) (Figure 1(b)). Compared with the UC group, DAI score was significantly decreased in the EGCG group (*p* < 0.05). In the sham group, the colonic surface of rats was smooth with no bleeding or ulceration. Intestinal hemorrhage, ulcer, and intestinal adhesions were seen in UC rats. After EGCG intervention, small ulcer foci were visible in the colonic tissue (Figure 1(a)). It was found that the score of colonic mucosal injury was significantly increased in the UC group and diminished after EGCG intervention (Figure 1(c)). Hematoxylin-eosin staining results demonstrated that mucosal epithelial cell degeneration/necrosis and inflammatory cell infiltration were observed in the UC group (Figure 1(d)). Mild epithelial cell degeneration/necrosis and small amounts of inflammatory cells were distinct in the EGCG group. Histomorphological score of mucosa in rats was significantly lower in the UC group than in the sham group. After intervention with TLR4 inhibitor, scores and HE staining were not significantly different compared with the UC group (*p* > 0.05).

### 3.2. EGCG Suppresses Inflammatory Response in UC Rats

**Figure 2:**
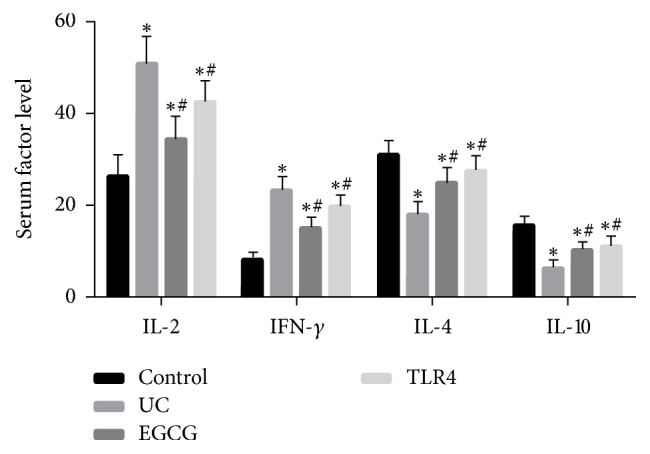
Peripheral blood was collected from the control group, UC group, EGCG group, and TLR4 group (*n* = 10), sera were isolated, and ELISA was used to detect the IL-2, IFN-*γ*, IL-4, and IL-10 levels. Compared with control group, ^*∗*^*p* < 0.05. Compared with UC group, ^#^*p* < 0.05.

Serum IL-2, IFN-*γ*, IL-4, and IL-10 levels were measured in UC rats with ELISA. Compared with the sham group, serum IL-2 and IFN-*γ* levels were higher (*p* < 0.05), but IL-4 and IL-10 levels were lower in the UC group (*p* < 0.05). After EGCG intervention, serum IL-2 and IFN-*γ* levels were decreased (*p* < 0.05), but IL-4 and IL-10 levels were increased (*p* < 0.05) (Figure 2). All data suggested that EGCG could improve inflammatory response in UC rats.

### 3.3. EGCG Improves the Th1/Th2 Balance in UC Rats

**Figure 3:**
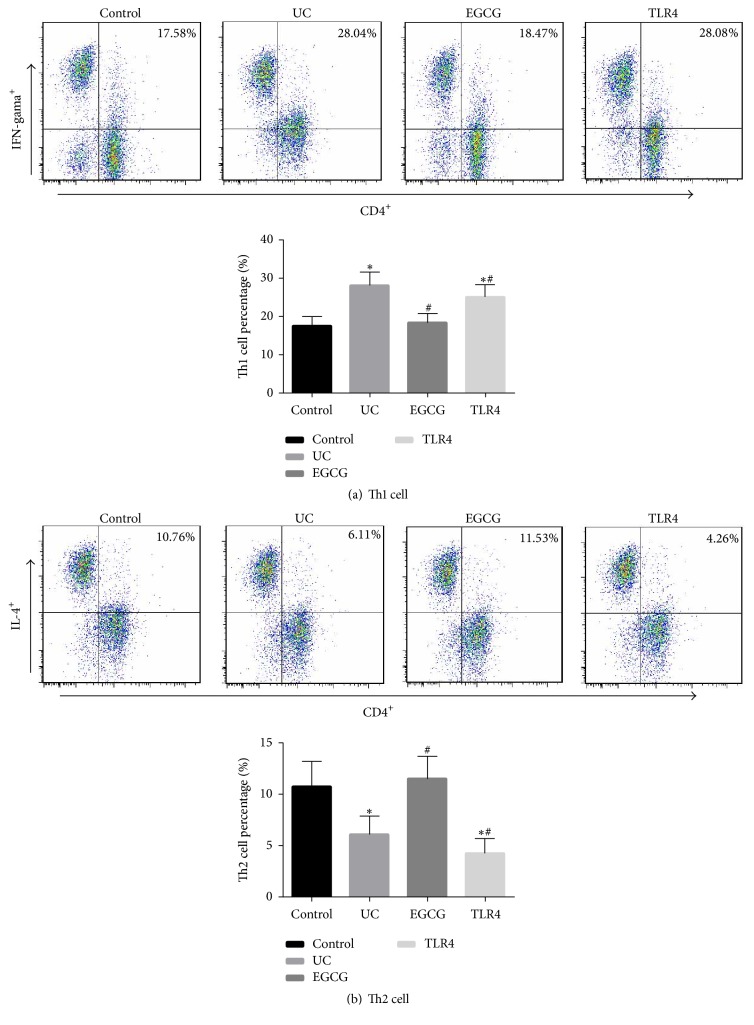
The colonic tissue was collected from UC group, EGCG group, and TLR4 group (*n* = 10), and PMBA was isolated from the colonic tissue. Flow cytometry was used to detect the CD4^+^IFN-*γ*^+^ (Th1) cell percentage (a). Different cell subsets were distinguished according to different cell labels. Flow cytometry was used to detect the CD4^+^IL-4^+^ (Th2) cell percentage (b). Different cell subsets were distinguished according to different cell labels. Compared with control group, ^*∗*^*p* < 0.05. Compared with UC group, ^#^*p* < 0.05.

To identify that EGCG can improve the Th1/Th2 balance, flow cytometry was utilized to analyze Th1 cells and Th2 cells in the rat colon. Data suggested that, compared with the sham group, IFN-*γ* expression in CD4^+^ T cells was significantly increased (*p* < 0.05) (Figure 3(a)), but IL-4 expression in CD4^+^ T cells was significantly reduced (*p* < 0.05) (Figure 3(b)). After EGCG intervention, Th1 cells and Th2 cells were approximately balanced. Compared with the EGCG group, the number of CD4^+^IFN-*γ*^+^ cells was higher in the rat colon (*p* > 0.05), but the number of CD4^+^IL-4^+^ cells was lower (*p* > 0.05) in the EGCG group after inhibiting TLR4 expression. These findings verified that EGCG could improve the Th1/Th2 balance in UC rats probably through TLR4/MyD88/NF-*κ*B signaling pathway.

### 3.4. EGCG Diminishes the Expression Levels of TLR4/MyD88/NF-*κ*B Pathway-Related Proteins in the Colon of UC Rats

**Figure 4:**
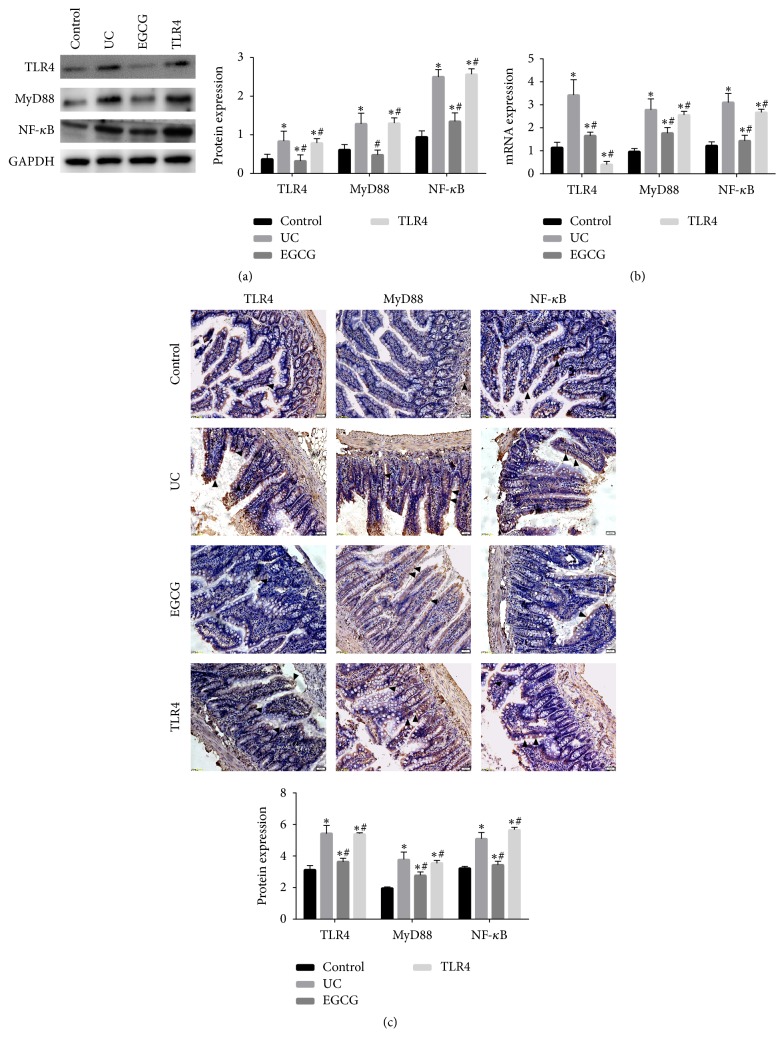
After establishing the UC model, EGCG and TLR4 inhibitor E5564 intervened. The colonic tissue was collected and the total protein was extracted. Western blot assay was used to detect the expressions of TLR4, MyD88, and NF-*κ*B protein expressions (a). The 18 colonic tissues were collected, the total RNAs were extracted, and the first strand of DNA was synthesized by reverse transcription. QRT-PCR was used to detect the expressions of TLR4, MyD88, and NF-*κ*B at mRNA level (b). The colon tissue was collected, fixed in 10% formaldehyde, embedded, and sliced into sections. Immunohistochemistry was used to detect the TLR4, MyD88, and NF-*κ*B expressions (c). Compared with control group, ^*∗*^*p* < 0.05. Compared with UC group, ^#^*p* < 0.05.

Compared with the sham group, the expression levels of TLR4, MyD88, and NF-*κ*B proteins were significantly higher in the UC group (*p* < 0.05). After EGCG intervention, TLR4, MyD88, and NF-*κ*B protein expression was significantly decreased (*p* < 0.05) (Figure 4(a)). After adding TLR4 inhibitor E5564, the protective effect of EGCG was weakened. TLR4, MyD88, and NF-*κ*B mRNA expression (Figure 4(b)) and immunohistochemistry (Figure 4(c)) supported this conclusion. All data verified that EGCG improved Th1/Th2 balance and suppressed colonic mucosal injury in UC rats through TLR4/MyD88/NF-*κ*B signaling pathway.

## 4. Discussion

UC is a chronic intestinal immune disease. UC can be alleviated by drug therapy, but it is easy to relapse and increase the risk of cancerization of colitis. This study established UC rat models and found that EGCG mitigated DSS-induced colonic mucosal injury, reduced epithelial cell degeneration/necrosis, and decreased inflammatory factor and apoptosis protein expression. Furthermore, EGCG could maintain Th1/Th2 balance and diminish DSS-induced MyD88 and NF-*κ*B protein expression in the colonic mucosa TLR4 pathway. Our results indicated that EGCG had protective effect on UC by modulating TLR4/MyD88/NF-*κ*B pathway.

EGCG is a major component in tea. EGCG has been shown to suppress inflammation, oxidation, tumor, and apoptosis. Katiyar and Mukhtar [18] found that EGCG significantly inhibited the activity of UVB-induced antioxidant enzymes and inhibited the oxidative stress induced by UVB. Brückner et al. [19] verified that EGCG suppressed the activity of oxygen free radicals in neutrophils [19] and exerted anti-inflammatory effects by scavenging free radicals and oxidants and inhibiting oxidative stress [20, 21]. We found that EGCG lessened the extent of DSS-induced colonic mucosal injury and epithelial cell degeneration/necrosis, and our results indicated that EGCG has protective effects on colon injury induced by DSS.

CD4^+^ T cells induced by IFN-*γ* and IL-2 differentiate into Th1 cells and mediate cell immunity [22]. CD4^+^ T cells induced by IL-4 differentiate into Th2 cells, secrete IL-4, IL-5, and IL-10 to activate B cells, and mediate humoral immunity and hypersensitivity [9]. The Th1/Th2 imbalance is associated with tumor immune escape, microbial infections such as bacteria and viruses, also involved in allergic diseases, autoimmune diseases, and transplant rejection, and plays an important role in mediating UC development [23–25]. To maintain intestinal balance, the normal intestinal mucosal immune system must maintain a balance between proinflammatory cytokines and anti-inflammatory cytokines or cytokine regulatory network. Once the balance is broken, it may cause excessive proliferation of the effector cells or regulate the decline of cellular function and aggravate the inflammatory response of the mucosa. Our study demonstrated that serum IL-2 and IFN-*γ* levels increased, but IL-4 and IL-10 levels decreased in UC rats, which suggested that it may be related to Th1/Th2 balance. Th1 cells and Th2 cells in the rat spleen were further analyzed by flow cytometry. Results demonstrated that the percentage of Th1 cells increased, but the percentage of Th2 cells diminished in the spleen of UC rats. After EGCG intervention, the percentage of Th1 cells decreased, but the percentage of Th2 cells increased, suggesting that EGCG improved the Th1/Th2 balance in rat intestinal mucosa, and promoted self-repair of intestinal mucosa.

TLR is an important component of innate immune recognition receptors and plays an important role in the identification of microorganisms in hospitals. TLR activates innate immune response, causes cytokine release, upregulates the expression of costimulatory molecules, and provides necessary activation signals for acquired immune response by identifying lipopolysaccharide, lipoproteins, and genetic material nucleic acids of microorganism in hospitals [26]. TLR4, an important component of TLR, can mediate the high reactivity of intestinal epithelial cells on cell wall components, activate NF-*κ*B, cause effector cells to secrete cytokines such as tumor necrosis factor-*α*, and play an important role in inflammatory response. Our results showed that TLR4, MyD88, and NF-*κ*B expression was high in UC rats. After EGCG intervention, TLR4, MyD88, and NF-*κ*B protein expressions were remarkably decreased. EGCG may exert the anti-inflammatory effect and regulatory effect on Th1/Th2 balance through TLR4/MyD88/NF-*κ*B signaling pathway. To further verify above results, we used TLR4 inhibitor E5564 to block the pathway and found that protective effect of EGCG disappeared; Th1/Th2 balance was broken; and the expression of TLR4, MyD88, and NF-*κ*B significantly decreased. These findings further confirmed that EGCG maintained Th1/Th2 balance and suppressed immunoinflammatory response in colonic tissue through TLR4/MyD88/NF-*κ*B signaling pathway.

In summary, EGCG can inhibit the intestinal immunoinflammatory response, reduce the severity of UC, and maintain the Th1/Th2 balance through the TLR4/MyD88/NF-*κ*B signaling pathway. This lays theoretical foundation for developing target therapy for UC.

## Figures and Tables

**Table 1:** qRT-PCR using gene primers.

Gene	Primer (5′ → 3′)
TLR4	Forward: TGAATCCCTGCATAGAGGTA
	Reverse: GACCGTTCTGTCATGGAAGG
MyD88	Forward: TACAAAGCAATGAAGAAGGA
	Reverse: TTGCATGAGGTAGTGGCACG
NF-*κ*B	Forward: CAGCCTGGTGGGCAAGCACT
	Reverse: GAAGGATTTGGGGACTTT
*β*-Actin	Forward: TCCTCACTGAGGCCCCGC
	Reverse: CTGCCCCATGCCATTCTC
